# Cancer stem cell secretome in the tumor microenvironment: a key point for an effective personalized cancer treatment

**DOI:** 10.1186/s13045-020-00966-3

**Published:** 2020-10-15

**Authors:** Julia López de Andrés, Carmen Griñán-Lisón, Gema Jiménez, Juan Antonio Marchal

**Affiliations:** 1 Biopathology and Regenerative Medicine Institute (IBIMER), Centre for Biomedical Research (CIBM), University of Granada, 18100 Granada, Spain; 2 Instituto de Investigación Biosanitaria Ibs.GRANADA, University Hospitals of Granada-University of Granada, 18100 Granada, Spain; 3 Excellence Research Unit “Modeling Nature” (MNat), University of Granada, Granada, Spain; 4 Department of Health Sciences, University of Jaén, 23071 Jaén, Spain; 5 Department of Human Anatomy and Embryology, Faculty of Medicine, University of Granada, 18016 Granada, Spain

**Keywords:** Cancer stem cells, Tumor microenvironment, Secretome, Growth factors, Interleukins, miRNAs, Exosomes

## Abstract

Cancer stem cells (CSCs) represent a tumor subpopulation responsible for tumor metastasis and resistance to chemo- and radiotherapy, ultimately leading to tumor relapse. As a consequence, the detection and eradication of this cell subpopulation represent a current challenge in oncology medicine. CSC phenotype is dependent on the tumor microenvironment (TME), which involves stem and differentiated tumor cells, as well as different cell types, such as mesenchymal stem cells, endothelial cells, fibroblasts and cells of the immune system, in addition to the extracellular matrix (ECM), different in composition to the ECM in healthy tissues. CSCs regulate multiple cancer hallmarks through the interaction with cells and ECM in their environment by secreting extracellular vesicles including exosomes, and soluble factors such as interleukins, cytokines, growth factors and other metabolites to the TME. Through these factors, CSCs generate and activate their own tumor niche by recruiting stromal cells and modulate angiogenesis, metastasis, resistance to antitumor treatments and their own maintenance by the secretion of different factors such as IL-6, VEGF and TGF-ß. Due to the strong influence of the CSC secretome on disease development, the new antitumor therapies focus on targeting these communication networks to eradicate the tumor and prevent metastasis, tumor relapse and drug resistance. This review summarizes for the first time the main components of the CSC secretome and how they mediate different tumor processes. Lastly, the relevance of the CSC secretome in the development of more precise and personalized antitumor therapies is discussed.

## Introduction

The cancer stem cell (CSC) model is based on the identification of tumor cells in different stages of differentiation in a wide variety of tumors, including ovarian [1], breast [2, 3], brain [4], lung cancer [5], melanoma [6], prostate [7], colorectal [8] and liver cancer [9]. All of them are composed by a small subpopulation of cells with stem cell-like characteristics such as quiescence, slow cell cycle, expression of embryonic SC transcription factors and epigenomic regulation driven by micro-RNAs (miRNAs) [10]. Like normal SCs, CSCs can self-renew and divide asymmetrically to give rise to daughter cells, which constitute the bulk of the tumor, and this makes CSCs are responsible for the maintenance and proliferation of the tumor, as observed in healthy tissues [11].

However, identification of these subpopulations has not been easy, and although several markers have been described, tumor heterogeneity and inter-patient variations make it difficult to define robust markers [12]. In general terms, the most commonly used indicators to identify CSCs are surface markers such as CD133 and CD44 [13, 14], increased activity of aldehyde dehydrogenase (ALDH) [14, 15] and their ability to exclude Hoechst 33342 (side population) [16], and to form spheres in vitro [17].

In addition, CSCs drive tumor drug resistance due to their ability to enter a quiescent state, activate DNA repair mechanisms, reactivate drug efflux system and protect against ROS [12], ultimately being responsible for disease relapse. Therefore, the CSC model explains the poor prognosis of the disease and indicates that identifying and attacking CSCs are currently a major challenge in cancer research.

As the importance of CSCs in tumor development has been elucidated, special attention has also been paid to their environment, since the tumor niche has a strong influence on the tumor behavior. The tumor microenvironment (TME) includes stem and differentiated cancer cells, the extracellular matrix (ECM), mesenchymal stem cells (MSCs), cancer-associated fibroblasts (CAFs), endothelial cells (ECs), immune system cells, and a complex network of cytokines and growth factors [18]. All these components orchestrate tumor processes in different ways. Non-tumor and differentiated tumor cells interact closely with CSCs by modulating their activity and contributing to key tumor processes such as tumor growth, metastasis, angiogenesis and immune system evasion [18]. Indeed, TME cells also promote resistance to antitumor therapies, since the secretion of soluble factors such as interleukin-6 (IL-6), hepatocyte growth factor (HGF), fibroblast growth factor (FGF), or transforming growth factor ß (TGF-ß) and ECM adhesion proteins such as integrins leads to the activation of several tumor survival pathways [19]. Additionally, the ECM has a different composition, organization and post-transcriptional modification in the TME than the surrounding normal tissue [20] and largely influences the intratumor signaling, transport mechanism, cell motility, metastasis and immune response [21, 22]. Moreover, tumor ECM shows higher density and stiffness, which can interfere on nutrient, oxygen and metabolite diffusion which in turn lead to tumor hypoxia. This stiff ECM also acts as a physical barrier to the action of chemo- and radiotherapy agents. Tumor hypoxia and the barrier capacity are related to poor treatment response [20, 23].

Importantly, CSCs do not merely adapt to the TME; they also form their own niches by recruiting and activating other cells and modify their environment in different ways [24]. Understanding the interaction of CSCs with their niche may be crucial to design effective cancer treatments and selectively target this cell subpopulation.

This review examines for the first time the main components secreted by CSCs to generate and modify their own environment and to orchestrate the tumor hallmarks. To this end, we describe the role played by CSC secretome in cell recruitment, in the interactions with tumor niche as well as in distal metastasis. Finally, the impact of CSCs on the development of resistance to current antitumor treatments and the new therapies that focus on overcoming these issues by targeting the CSC secretome are also discussed.

## Cancer stem cell secretome

**Fig. 1:**
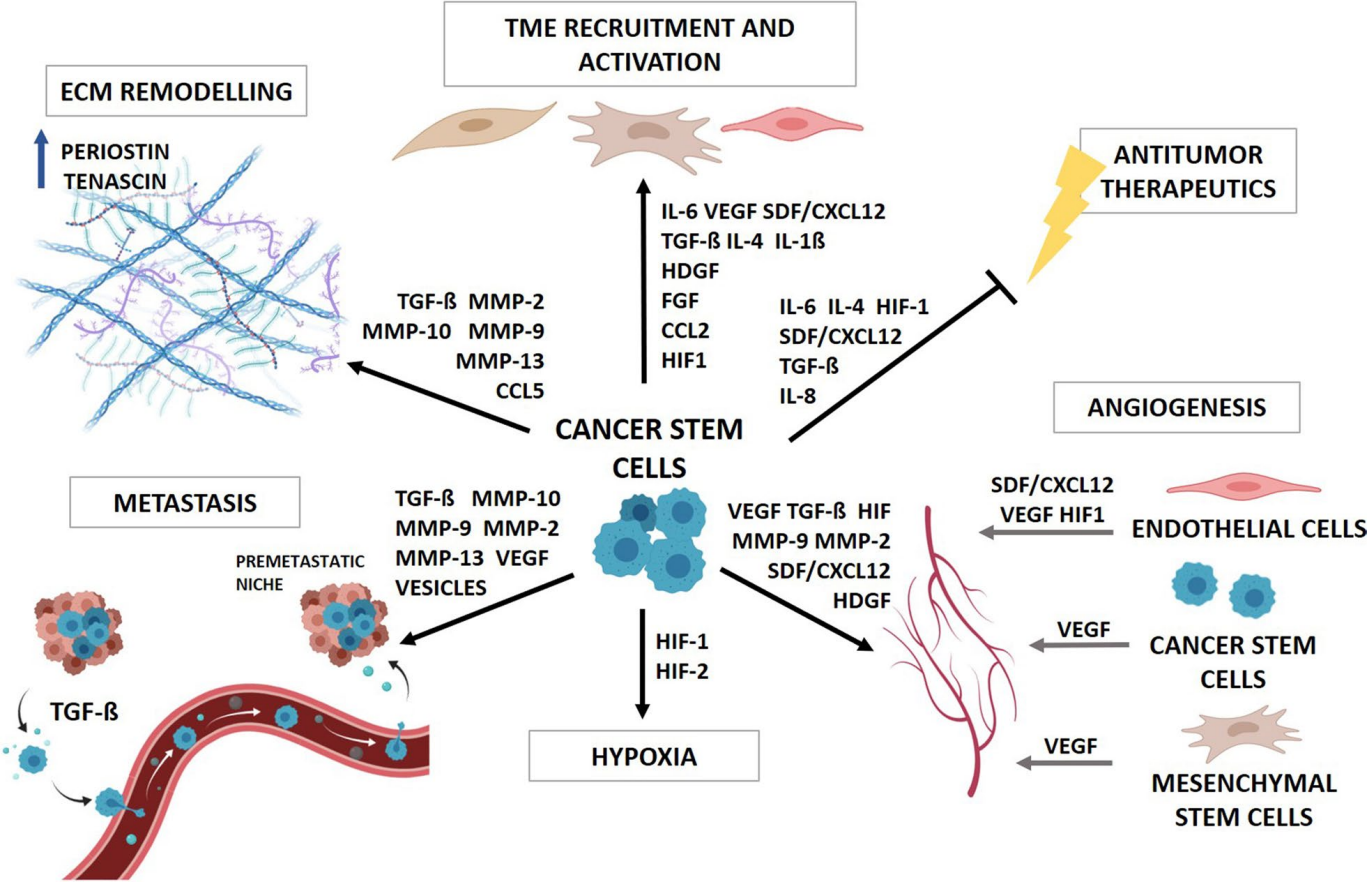
Crosstalk between CSCs and TME components. CSCs secrete a wide variety of soluble factors to recruit and activate stromal cells and reorganize the ECM, as well as to promote angiogenesis, metastasis, hypoxia, immune evasion and tumor progression. These factors also regulate their own maintenance and tumor niche maintenance and the response to different anti-tumor therapies

Secretome refers to all the molecules secreted by a cell or shedded from its membrane and is fundamental for cell–cell communication. Despite that the secretome has commonly been defined only by the protein fraction, the non-protein components such as lipids, miRNAs and messenger-RNAs (mRNAs) isolated in or secreted via vesicular bodies have been also incorporated into this definition [25]. There is increasing evidence that CSCs regulate different tumor hallmarks such as angiogenesis, tumor growth, metastasis, drug resistance and immune dysregulation through their secretome (Fig. 1). CSCs communicate with the TME by releasing microvesicles (MVs) and exosomes, as well as a wide range of soluble factors including chemokines, cytokines, growth factors, hormones and metabolites [26]. MVs differ from exosomes in size (MVs range from 50 to 10,000 nm while exosomes are typically 30–150 nm in diameter), their secretion mechanism and cargo [27].

Some factors involved in the communication of CSCs with their environment such as IL-6, IL-8 and IL-1ß, vascular endothelial growth factor (VEGF) and various matrix metalloproteinases (MMPs) can be released free into the extracellular space or encapsulated in exosomes and MVs [28–30]. In addition, various cytokines such as CCL2 or CCL5 [31], TGF-ß [32–34] and hypoxia-inducible factor 1 (HIF-1) can also be released via exosomes to the TME [35]. All these factors perform multiple functions within the tumor, since they promote the formation and activation of the TME, hypoxia, tumor vascularization and metastasis, or chemo- and radiotherapy resistance, as it will be discussed in more detail below (Fig. 1).

In addition, several studies have shown that not only soluble factors but also miRNAs strongly contribute to cancer development and progression by altering the secretome, promoting both tumorigenic and tumor suppression responses. MiRNAs are small RNAs molecules of 18–22 nucleotides with the ability to regulate cancer-related processes including cellular proliferation, cell cycle arrest, senescence, DNA damage response, apoptosis, metastasis and CSC properties. These diverse functional features are tumor and tissue specific and can have a positive or negative effect, and changes in miRNA levels may influence their function [36–38]. One recent finding is that miRNAs can be found inside exosomes or MVs and prevent RNase degradation. Therefore, these exosome-transferred miRNAs have emerged as a new mechanism mediating the tumor–stroma crosstalk and metastasis [31, 39, 40] (Table 1). Therefore, it is clear that miRNAs secreted by CSCs could be potent regulators of the secretome involved in cancer initiation and progression. For example, miR-200 family has been shown to inhibit the epithelial–mesenchymal transition (EMT) and enhance the reverse process [41, 42].

**Table 1:** Exosomes or extracellular vesicles-derived miRNAs from cancer stem cells

miRNAs	Cancer type	Functions	References
miR-10b, miR-105, miR-9 miR-195, miR-203a miR-200 family	Breast cancer	Invasiveness, endothelial cell migration, angiogenesis and metastasis	[39, 43–45]
miR-21, miR-34, miR-155	Oral cancer	Proliferation, migration and poor prognosis	[44]
miR-19b, miR-29c, miR-151	Renal cancer	EMT and metastasis	[46, 47, 48]
miR‐215 and miR‐375, miR‐17–92 cluster, miR‐200c	Colorectal cancer	Relapse and poor prognosis, tumor development and metastasis	[47]
miR-21	Glioblastoma	Angiogenesis and tumor growth	[36, 50]
miR-139, miR-183	Prostate cancer	Cell proliferation and migration	[48]
miR-21, miR-221 miR-146, miR-17, miR-155	Pancreatic cancer	Angiogenesis, tumor growth, metastasis and migration and invasion in advanced tumor stages	[36, 49, 51]

## Secretome in the interaction with stromal cells

**Fig. 2:**
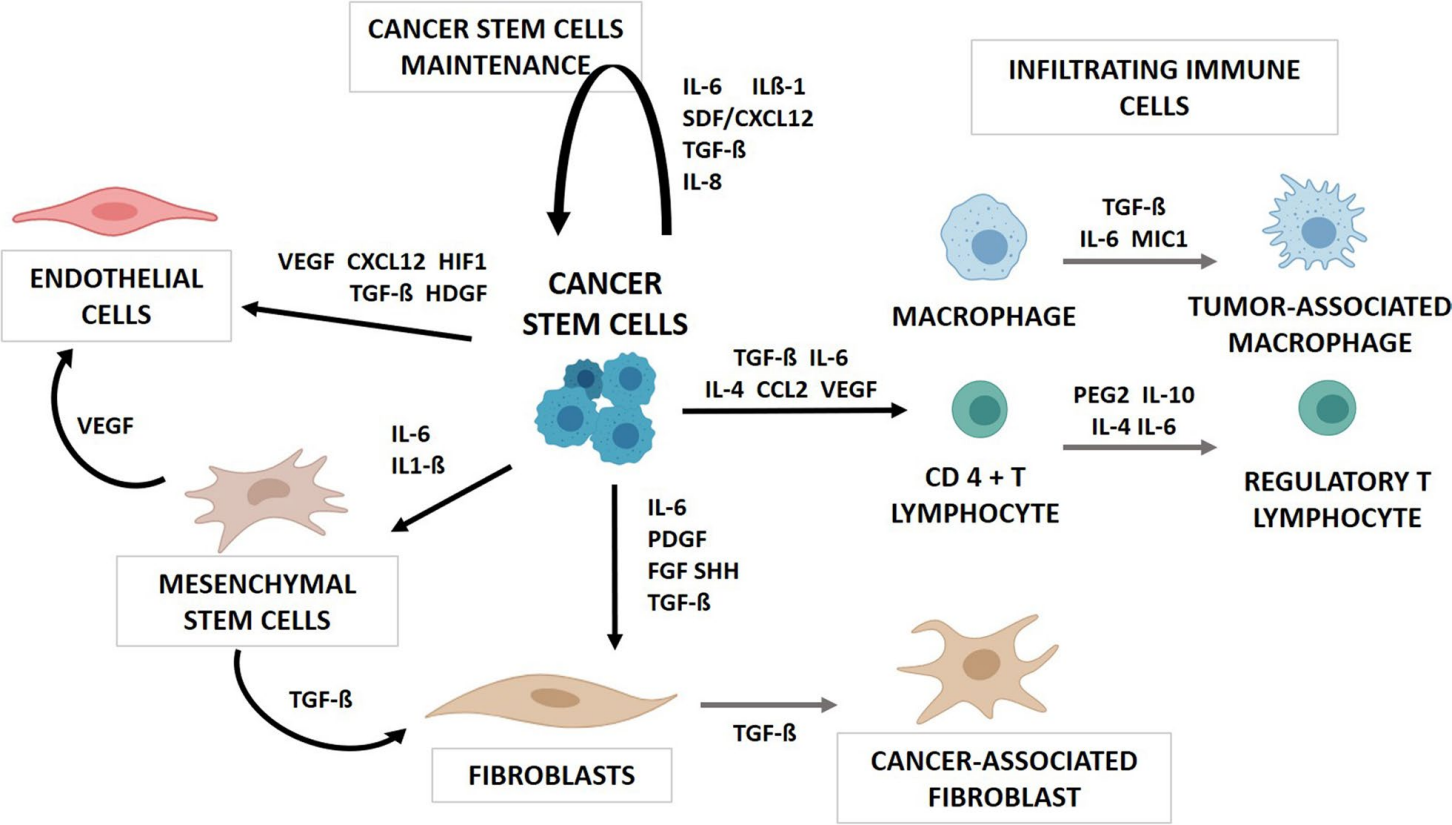
Crosstalk between CSCs and TME cells. CSCs secrete different factors in order to recruit and activate mesenchymal stem cells (MSCs), endothelial cells (ECs), cancer-associated fibroblast (CAFs) and infiltrating immune cells (IICs) to the TME. CSCs also promote their own maintenance and are therefore able to regulate processes of hypoxia, vascularization, metastasis and immune response evasion

CSCs play an essential role in tumor niche generation by recruiting and activating TME cells through different signaling pathways [41] (Fig. 2). In fact, many of these pathways result on a communication loop between CSCs and stromal cells, whereby CSCs self-regulate and regulate their environment.

### Mesenchymal stem cells (MSCs)

Mesenchymal stem cells perform key functions in the development of cancer including regulation of inflammatory processes, angiogenesis, metastasis, maintenance of CSCs and tumor growth [50, 51]. For this reason, MSCs are recruited into the tumor niche by CSCs and interact with each other through a wide network of cytokines [52] (Fig. 2).

First, CSCs recruit MSCs to the sites of primary tumor growth by secreting IL-6, which also triggers other responses in the tumor niche. In turn, IL-6 induces the production of CXCL7 by MSCs [52], which has been shown to promote tumor invasiveness and metastasis in murine models [53, 54], as well as tumor growth through interaction with the tumor receptor CXCR2 [53] that, in turn, induces the synthesis of other cytokines including IL-6 and IL-8.

Moreover, CSCs in pancreatic cancer have been found to overexpress IL-1ß [55] to attract MSCs by promoting MMP-1 secretion, which in turn activates the protease-activated receptor 1 (PAR1) and G-protein-coupled signal pathways, resulting in migration and recruitment of MSCs to the tumor niche. Moreover, tumor-secreted IL-1ß induces the expression of several chemokines by MSCs [56, 57] because it promotes the expression of nuclear factor kB (NF-kB), a major regulator of chemokine expression [57]. As with IL-6, IL-1ß also interacts with other cell types and triggers other pathways in the TME directly affecting tumor growth, invasion and angiogenesis [56], by inducing the secretion of angiogenic factors by tumor and stromal cells. However, it seems that the effect of IL-1ß is related to tumor type and the TME, since a negative effect on IL-1ß-mediated tumor growth has also been reported, explained by an increased immune response [58, 59].

Finally, glioma CSCs have been shown to secrete stromal cell-derived factor 1 (SDF-1/CXCL12) in order to recruit MSCs [60]. MSCs also secrete SDF-1/CXCL12, and communication between CSCs and MSCs through SDF-1/CXCL12 leads to tumor progression through different ways, including CSs survival, tumor growth, metastasis and angiogenesis processes [61, 62].

### Cancer-associated fibroblasts (CAFs)

Cancer-associated fibroblasts (CAFs) are also key actors in TME supporting tumor maintenance, angiogenesis, EMT and metastasis and producing ECM components [63]. CSCs recruit CAFs either by activating adjacent fibroblasts, CAFs or transforming MSCs through the secretion of different factors like platelet-derived growth factor (PDGF), FGF, IL-6 and TGF-ß [24, 64–66] (Fig. 2). In breast cancer, it has also been described that the activation of fibroblasts to CAFs requires the activation of STAT3 by these cytokines, which results in CAFs showing higher CCL2 expression than normal fibroblasts, which induces Notch1 expression of CSCs and, therefore, promotes stemness maintenance in a bidirectional interaction [67]. On the other hand, CSCs can induce differentiation of MSCs into CAFs through the secretion of TGF-ß via the activation of the TGFBR1/Smad pathway [34, 68, 69].

Furthermore, in a recent study using a murine model of breast cancer, CSCs have shown to produce the Hedgehog (Hh) ligand Sonic Hedgehog (SHH), activating the Hh signaling pathway in CAFs, which leads to increased CAF proliferation and ECM deposition and enhances the production of other factors such as ACTIVIN A, insulin-like growth factor 1 (IGF-1) and leukemia inhibitory factor (LIF), which result in CSC growth and self-renewal [70], in a feedback communication between CAFs and CSCs.

### Immune cells

In the TME, immune system cells may exhibit tumorigenic or antitumor activity depending on environmental signals [71]. For this reason, the communication with tumor cells becomes an important factor. The role of CSCs is not only to recruit and activate cells in the tumor niche, but also the evasion of the immune response (Fig. 2). CSCs recruit macrophages to the tumor niche by producing pro-inflammatory cytokines and chemokines, such as CCL2 [72], IL-6, IL-4, VEGF and TGF-ß [73, 74]. Once in the TME, macrophages are activated to tumor-associated macrophages (TAMs) through CSC-secreted factors such as IL4, TGF-ß and macrophage inhibitory cytokine 1 (MIC-1), which also inhibits the phagocytic activity of macrophages [75, 76]. Moreover, CSC-released TGF-ß [77, 78] induces the differentiation of CD4 + T lymphocytes to regulatory T lymphocytes (Treg) by stimulating the synthesis of FOXP3, thereby having an immunosuppressive effect on the tumor [79, 80]. However, this is not the only pathway used by CSCs for macrophage activation [81, 82]. Treg causes a decrease in tumor immunity not only by regulating the accumulation of T lymphocytes, but also by releasing other factors with immunosuppressive roles such as TGF-ß and IL-10 [83].

A major hallmark of CSCs is to evade the immune response [84] through an inadequate antigen presentation [82, 85, 86] and the ability to create a differentiated tumor cell barrier around them [87]. Additionally, CSCs secrete different molecules with protective function against both innate and adaptive immune responses such as TGF-ß, IL-4, IL-6, IL-10 and prostaglandin E2 (PGE2) [73, 80, 88]. In addition, FOXP3 in Treg, whose expression is regulated by TGF-ß and other factors, also inhibits the secretion of IL-2, interferon gamma and IL-4 [89]. Furthermore, CSCs secrete exosomes with an immune response modulating effect by suppressing T-cell response [90] and inhibiting dendritic cell differentiation [91]. These finding show that CSCs are capable of triggering multiple pathways for immune evasion.

In summary, the CSC secretome is responsible for the recruitment and activation of MSCs, CAFs and immune cells to the TME. In addition, they promote the function of these cell types, thus leading the regulation of inflammatory responses, tumor growth, angiogenesis, metastasis and their own maintenance.

## Secretome in angiogenesis

Angiogenesis is a central process that promotes tumor development and metastasis. CSCs modulate the vascularization of the tumor niche mainly through VEGF, but other factors are also involved (Fig. 2). CSCs recruit endothelial cells (ECs) to the tumor niche and induce angiogenesis by secreting HIF-1, VEGF and SDF-1/CXCL12 [92–94]. Furthermore, MSCs migrate to the tumor niche recruited by CSCs, and once there they differentiate into ECs through the action of VEGF [95–97]. In addition, CSCs are capable of differentiating into ECs and endothelial progenitor cells (EPCs) and form vessel-like networks in a process called “vascular mimicry,” mediated also by VEGF [98–101]. Indeed, CSCs preferentially overexpress more VEGF receptors (VEGFR-1 and VEGFR-2) than their differentiated counterparts and their activation by VEGF mediates chemotaxis, tubule formation and vascular marker expression [100–103]. Likewise, CSCs show high expression of vascular–endothelial cadherin (VE-Cadherin) and Notch, both involved in the transformation to ECs and EPCs [102–106], as well as MMP-2 and MMP-9, which promote ECM remodeling thus promoting new vessel formation by CSCs [105, 106]. Moreover, CSCs overexpress CXCR4, whose SDF-1/CXCL12 ligand induces VEGF production via activation of the P13K/AKT signaling pathway [107, 108]. This makes that stromal cells and CSCs can also modulate angiogenesis by promoting the expression of CSC-secreted VEGF [109]. CSC-secreted VEGF plays other critical roles in the tumor niche since it enhances CSCs proliferation by stimulating neuropilin-1, a co-receptor of VEGFR2, and thus promotes tumor progression and relapse [110–112].

Furthermore, CSCs secrete TGF-β [77, 78] that can also induce the expression of VEGF and another angiogenic factor, the connective tissue growth factor (CTGF), in both epithelial cells and fibroblasts [113, 114]. Finally, a comparative study of glioma CSCs and non-SCs secretome has identified an increase in hepatoma-derived growth factor (HDGF) in glioblastoma stem-like cells (GSCs) linked to angiogenesis in vivo [115].

Taken together, these findings demonstrate that the CSC secretome modulates the generation of new vasculature in the TME by recruiting ECs and MSCs. In addition, the secretome involved in this process also enhances the maintenance of CSCs and therefore tumor proliferation and relapse.

Nonetheless, the vascular network formed to support the rapid tumor growth is aberrant, with disorganized, immature and highly permeable blood vessels, and cannot fully fulfill its functions [116]. This limits tumor perfusion, decreases oxygen supply and increases hypoxia in the tumor and prevents the arrival of immune system cells. Poor perfusion also reduces the efficacy of radiation therapy and antitumor drug perfusion to the tumor, which allows tumor survival [116]. Furthermore, the high permeability, related to reduced coverage of the pericytes and their binding to the ECs, facilitates the metastatic spread of cancer cells, mainly CSCs [117].

## Secretome in hypoxia

The rapid proliferation of cancer cells and the TME aberrant vasculature cause hypoxic regions within the tumors. In response to this situation, both CSCs and non-SCs secrete HIF-1, which is stabilized only in areas with very low oxygen level [118, 119]. HIF-1 in the tumor niche is related to poor prognosis because it enhances tumor spread and CSC self-renewal [15, 120–122], promoting the stem phenotype by enhancing Notch-Hes1 or ALDH expression. Furthermore, in several tumor types HIF-1 has been reported to promote resistance to therapy [23] and EMT phenotype [123], to remove or prevent toxic metabolic waste products [118, 124]. HIF-1 is also involved in angiogenic processes by inducing VEGF and VEGFR2 expression [125–127] and can suppress antitumor immune responses [128].

On the other hand, the hypoxia-inducible factor 2 (HIF-2) and HIF-regulated genes are preferentially expressed in CSCs compared to their differentiated counterparts, which is associated with poorer prognosis and higher CSCs proliferation and survival [129]. Furthermore, HIF-2 has been shown to be expressed at low levels of hypoxia and even at physiological levels of oxygen [119, 129]; therefore, its function is not limited to hypoxic regions.

Moreover, the secretome of hypoxic tumor environments regulates miR-210 expression in a positive way, as shown by the fact that HIF-1 secreted by both CSCs and non-SCs promotes its expression. MiR-210 is an important mediator of the response to low oxygen tension and promotes mRNA degradation of normoxic genes in several cancers, particularly in pancreatic cancer. Thus, circulating miR-210 levels could serve as a useful biomarker for diagnosis in cancers with extremely hypoxic signatures [130, 131]. In addition, tumor-secreted HIF-1 induces miR-155 expression under hypoxic conditions and plays a dual role in maintaining this factor as miR-155 directly targets HIF-1 and suppresses its expression and miR-155 suppresses the translation of von Hippel–Lindau tumor suppressor (VHL) leading to increased HIF activity. This role is, however, not so contradictory, if we consider that miR-155 targets HIF-1 but not HIF-2α, which is more oncogenic. MiR-155 is also related inflammation since IL-6 induces its expression and this in turn activates the JAK2/STAT3 pathway, thus promoting tumor inflammation [132].

In conclusion, the CSC secretome regulates the response to hypoxic environments through HIF and miRNAs and its overexpression is related to poor prognosis, related to the effects on tumor maintenance and CSCs themselves.

## Secretome in CSC maintenance

In addition to interacting with stromal cells, CSCs regulate their maintenance in the tumor niche, as described above. In addition, CSCs regulate their self-renewal through the autocrine secretion of TGF-ß, which promotes stemness properties of CSCs in breast [133], colon [134] and ovary cancer [135] and induces self-renewal and prevents differentiation in glioma CSCs [136–138]. Indeed, CSCs secrete nodal and activin, from the TGF-ß family, linked to the CSCs self-renewal in pancreatic cancer in vitro and CSCs tumorigenicity in vivo [139]. Signaling pathways involved in TGF-ß-induced CSC phenotype acquisition include SMADs, AKT, SOX and MAPK, which in turn synergize with other signaling pathways to promote tumor invasion and metastasis [140].

Furthermore, CSC survival is promoted by autocrine production of different interleukins. In colon cancer, CSC-secreted IL-4 promotes their maintenance and inhibits apoptosis [76]. IL-1ß also promotes tumor growth and invasion by activating CSC self-renewal and EMT [56, 141]. IL-6 can induce tumor cell dedifferentiation in breast [142, 143], colon [144, 145], and prostate [146] cancers and regulates stemness in ovarian CSCs driven by ALDH1A1 expression [147]. Similarly, overexpression of IL-8 promotes pancreatic CSC self-renewal via IL-8/CXCR1 axis [148]. This is consistent with other studies, showing that the IL-8/CXCR1 pathway is essential for CSCs survival in breast cancer [149].

CSCs promote their maintenance and tumorigenicity through the secretion of exosomes. For example, GSCs secrete exosomes carrying chloride intracellular channel protein 1 (CLIC1), which affects tumor proliferation both in vivo and in vitro [150]. In pancreatic and colorectal cancer, exosomes released by CSCs stimulate the stemness phenotype and cell invasion and mobility [151]. In addition, CSCs deregulate miRNAs, which also affect their own maintenance. MiR-302/367 cluster was found to be strongly expressed in CSCs and strongly repressed during differentiation in most cancers, with its expression being highly correlated with the expression of CSC markers [152]. Interestingly, Rahimi et al. isolated CSCs using their stem cell-specific miR-302 expression and maintained CSCs stemness by continued selection [153]. Lastly, in colorectal cancer miR-146a increases the symmetrical division of CSCs, and miR-1246, one of the most differentially expressed miRNAs in the CSC population, is involved in self-renewal processes, tumorigenicity and drug resistance [36, 154].

Given the importance of CSC maintenance in the TME, the CSC secretome plays a key role on tumor development by promoting CSCs self-renewal, preventing their differentiation and even promoting the dedifferentiation of tumor cells to SCs.

## Secretome in extracellular matrix remodeling

ECM remodeling is essential for angiogenesis, metastasis and tumor growth, and since the ECM is a reservoir of many factors, its degradation results in their release into the environment. ECM remodeling occurs principally through the activity of matrix metalloprotease-10 (MMP-10), which enhances EMT, metastasis and CSC state [155, 156]. It has been reported that CSCs overexpress MMP-9 [157, 158], which allows the activation of inactive TGF-ß in ECM [159], MMP-2 and MMP-13 [105, 160], all related to increased metastatic and angiogenic capacity. In ovarian cancer cell lines, CSCs overexpress CCL5 and its CCR1, CCR3 and CCR5 receptors compared to their differentiated counterparts, resulting in increased autocrine-mediated invasion capacity by NF-kB activation and the consequently elevated MMP-9 secretion [161].

In addition, CSCs induce the production of certain ECM components such as periostin and tenascin through different factors, with TGF-ß playing a central role. That ECM components promote metastatis and support SCs functions [162–166]. Periostin promotes the acquisition of a stemness phenotype in tumor cells when it binds to Wnt ligands [167, 168]. Periostin is also essential for metastatic colonization with infiltrating tumor cells being able to induce periostin expression in the metastatic niche [166]. This protein can also increase the expression of the VEGFR by ECs [169]. Lastly, tenascin is involved in vascular mimicry by enhancing the release of MMP-2 and MMP-9 by CSCs [170]. Thereby, both ECM components are important for tumor angiogenesis.

CSC secretome is involved both in ECM degradation facilitating tumor metastasis, angiogenesis and proliferation, and in the generation of specific components involved in tumor behavior.

## Secretome in metastasis

Metastasis is a process that involves the dissemination of cells through lymphatic or blood vessels from the primary tumor to distant sites where colonization leads progressively to the growth of a secondary tumor. The EMT is a fundamental process for tumor invasion and involves the loss of epithelial properties and acquisition of motile and invasive phenotype [171]. The CSC secretome induces EMT and promotes metastasis in different ways. A key factor in the metastatic process is TGF-ß, which correlates with the expression of SC markers in tumor cells and promotes that phenotype, as described above, through induction of EMT [77, 137, 172–174]. TGF-ß has the ability to promote cell invasion and metastasis of CSCs and is not only expressed by CSCs, but also by other TME cells such as TAMs or CAFs [158, 175, 176]. However, TGF-ß is a ubiquitous cytokine that is expressed in nearly every cell type and therefore has an active role in various cellular processes. Although TGF-ß has been reported to promote tumor progression, invasion and metastasis in late-stage tumors, it has also been shown to act as a tumor suppressor as it can inhibit cell proliferation, induce apoptosis and mediate tumor cell differentiation in early-stage tumors. The role of TGF-ß depends on tumor stage and is regulated by tumor cells, stromal cells and the immune system. The mechanisms behind the dual role of TGF-ß are related to mutations in some components of the signaling pathway, but it may also be that there is no alteration of the tumor suppressor pathway, if not that it is inhibited [177, 178]. Several factors can promote tumor development including: (1) p53 mutations that activate the formation of the Smads2/3 and p63 complex that suppresses the action of p63 allowing TGF-ß to promote EMT; (2) loss of Smad4 function secondary to genetic alterations; (3) overexpression of Six1 (pro-metastatic regulator); (4) oncogenic activation of the Ras-RAF-MAPK pathway; (5) hypomethylation of the PDGFβ gene; and (6) DAB2 epigenetic downregulation [178]. Moreover, miR-106b-25 inhibits p21 and Bim (pro-apoptotic factor) and is probably involved in the positive regulation of Six1. Furthermore, miR-106b-25 can activate the tumorigenic path by inhibiting suppression growth through TGF-ß and repressing the inhibitory protein Smad7 [177].”

In addition, different miRNAs play a fundamental role in metastasis. Down-regulated miR-200 and let-7 miRNAs in CSCs can regulate EMT stem-like transition, self-renewal and metastasis in breast, prostate and colon cancer, and miR-34a downregulation in CSCs is related to self-renewal and asymmetric division [43, 152, 154, 179, 180]. miR-21 is a known “oncomiR” differentially up-regulated or over-expressed in CSCs and has been related to metastasis, poor prognosis, cell cycle and CSC promotion in many cancers. miR-221, miR-100, miR-10b or miR-125a upregulation in CSCs and non-CSCs can modulate breast CSC properties enhancing their invasion and migration potential [36, 152, 181]. Moreover, in metastatic breast cancer cells, exosome-secreted miR-10b, miR-105 and miR-9 are related to enhanced invasiveness through increased endothelial cell migration, angiogenesis and vascular permeability [43]. The miR-200 family found in extracellular vesicles of breast CSCs was related to their metastatic capacity [44]. In glioblastoma, miR-10b is upregulated in CSCs and its inhibition strongly reduces CSCs proliferation and metastasis. OncomiR-138 has also been identified as a prognostic biomarker of GSCs [180, 182]. In ovarian cancer, miR-5703, miR-630, miR-1246 and miR-320b were significantly dysregulated in CSCs compared with primary cancer cells, whereas miR-424-5p level was lower and associated with distant metastasis [183]. Finally, exosomes isolated from oral CSCs displayed nearly consistent downregulation of miR-34 and the up-regulation of miR-21 and miR-155 was related to increased CSC proliferation and migration; therefore, they may be consider indicators of poor prognosis [184]. However, miR-155 overexpression has been correlated with better prognosis and lower metastatic capacity [185, 186] and miR-21 appears down-regulated in CSCs [44]. These differences could be due CSC and inter-patient heterogeneity and to the dual role played by the same miRNA depending on cell state. This encourages further studies to elucidate the role of miRNAs based on this heterogeneity and its relationship with cancer progression. Lastly, miRNAs could be used as prognostic and predictive biomarkers of response to treatment.

Successful metastasis requires a favorable environment for the colonization and growth of tumor cells at the site of the secondary tumor, called the premetastatic niche (PMN) [187]. This PMN requires the recruitment and activation of local resident cells, alteration of the existing vasculature, ECM remodeling, as well as immune system deregulation [187, 188]. Several factors secreted by CSCs are involved in PMN formation. For example, VEGF increases vasculature permeability in the PMN [189] and stimulates MMP-9 expression in premetastatic tissue, thus facilitating tumor cell invasion [190]. Moreover, VEGF enhances the recruitment of bone marrow-derived cells (BMDCs), which are critical for PMN formation [191, 192] and facilitate tumor-promoting microenvironment through CCL9 secretion, induced by TGF-ß signaling [193]. In addition, TGF-ß and VEGF promote the expression of different inflammatory chemoattractants in the PMN [194]. Similarly, the CXCL12/CXCR4 axis is closely related to angiogenesis, proliferation, invasion and metastasis in most tumors by CXCR4 activation and migration of cells toward CXCL12. In addition, CXCL12 protein levels are highest in organs that are common sites of metastasis [61, 195–197]. CXCL12 induces MMP expression in cancer cells and up-regulates the activity of MMPs in tumor microenvironment, which promotes tumor invasion and metastasis [61]. CSCs express high levels of CXCR4 that have the ability to originate, maintain, disseminate and colonize metastasis sites and PMNs. CXCL12/CXCR4 signaling activation can be indicative of the metastatic CSC population [195, 198]. CSC migration is directed by CXCR4/CXCL12, playing a central role in chemotactic gradient perception. This signaling pathway is related with cell stemness and mobility [199]. Furthermore, intratumor hypoxia at primary site promotes PMN formation in secondary organs through enhancement of the expression of several factors and the involvement of exosomes [200, 201]. The role of exosomes and MVs from the primary tumor in the communication with PMN cells and modification of ECM has also been widely described [202–205]. Therefore, CSCs may play an important role in PMN formation, but further research is needed to clarify the specific role of the CSC secretome.

In summary, CSC secretome promotes metastasis by increasing EMT induction, tumor invasiveness, angiogenesis and CSC self-renewal. In addition, several secreted factors and vesicles are related to PMN formation, which make CSC secretome essential for successful metastasis.

## Secretome in chemoresistance

A major challenge in antitumor therapy is to effectively eradicate CSCs, ultimately responsible for tumor relapse after chemotherapy. CSCs show intrinsic resistance to drugs related to ABC transporter overexpression, high ALDH activity, apoptosis evasion mechanisms, enhanced DNA damage repair capacity and activation of key signaling pathways [24, 206–209]. Some of these mechanisms are regulated by CSC-secreted factors. HIF1, secreted by both CSCs and non-SCs, has been described in leukemia and lung tumors to promote radio and chemoresistance in hypoxic microenvironments by upregulating IGF1 expression and activating IGF1 receptor (IGF1R) [122, 210], which leads to an increase in the SC population and enhances EMT [211].

Furthermore, other interleukins with diverse roles in tumor behavior are also associated with increased drug resistance of CSCs. In colon cancer, the autocrine expression of CSC-secreted IL-4 promotes apoptosis evasion mechanisms, and treatment with IL-4 antibody especially sensitizes this subpopulation, promoting the efficacy of standard chemotherapeutic drugs [76]. Consistent with these studies, the chemoprotective action of IL-4 by increasing anti-apoptotic proteins Bcl-2 and Bcl-xL levels has been proved in other tumor types [212, 213]. The chemoprotective effects of IL-6 have also been described in breast CSCs, where HER2 overexpression increases IL-6 production [214]. Treatment with trastuzumab (target HER2 antibody) in breast cancer results in tumor chemoresistance related to the inactivation of the PTEN tumor suppressor gene. This is mediated by the IL-6 inflammatory loop activation, leading to the expansion of the CSC population. Furthermore, this CSC population secretes IL-6 to a much greater extent than non-SCs, which leads to a feedback loop that can be interrupted by an IL-6 receptor antibody, reducing the CSC population, tumor growth and metastasis [215]. A similar response to treatment with paclitaxel has been described in triple-negative breast cancer (TNBC) through the increase in the CSC population and tumor-initiating capacity in vivo. After treatment, high autocrine TGF-ß signaling and TGF-ß -dependent IL-8 overexpression occur, which promotes the potential of chemotherapy-resistant CSCs; therefore, this resistance can be reduced by pharmacological inhibition of TGF-ß [216]. Indeed, another study focused on blocking the IL-8 receptor CXCR1 using repertaxin, which selectively depleted CSC population in human breast cancer lines [149], confirming the role of this interleukin in maintaining the tumor stem population.

Moreover, the interaction of CSCs with TME cells also promotes a protective environment against chemotherapeutic agents. For example, both CSCs and MSCs secrete SDF-1/CXCL12, which interacts with their receptor CXCR4 overexpressed in CSCs [107, 217, 218]. This interaction contributes to the resistance of the tumor cells to chemotherapy-induced apoptosis [217, 219]. In fact, multiple trials with CXCR4 inhibitors have been conducted in solid tumors with promising anti-tumor effects [220]. Furthermore, another pathway that confers drug resistance to CSCs is their ability to influence cells of the immune system. CSCs from chemoresistant tumors were found to produce multiple proinflammatory cytokines (such as IL-1ß, IL-6, IL-8 or tumor necrosis factor) that also act to generate tumorigenic myeloid cells [221].

In addition to these strategies, the EMT has also been postulated as a therapeutic target against CSCs for its ability to promote chemoresistance through EMT-related signaling pathways and EMT transcription factors such as TGF-ß/Smad4, Hedgehog and Wnt [222, 223]. There are multiple examples of chemoresistance via EMT promotion such as doxorubicin resistance by upregulation of ABC transporters [224–227] or other apoptotic drugs such as cisplatin, paclitaxel and trastuzumab [228–231], which strongly supports the development of new therapies targeting EMT.

The epigenetic control of chemoresistance has been extensively described in CSCs addressing the classic CSC signaling pathways and the expression levels of genes related to chemoresistance and growth factor receptors and others [232]. Unfortunately, there is no research on the epigenetic control of secretome-associated chemoresistance in CSCs or in the total pool of the tumor population. Only one recent publication has related the inhibition of the HSP90 chaperone with a higher sensitivity to chemotherapy and a lower release of various cytokines (IL-8 and others) and, more interestingly, the HSP90 chaperone affected the survival of chemoresistant ALDH cell subpopulations [233]. In addition, there is little information available on oncogenic mutations associated with the influence of chemoresistance on CSC secretome, but p53 mutations have been reported to induce the release of an altered secretome by the tumor pool subpopulations that affects chemoresistance and other tumor processes [234, 235]. For this reason, the epigenetic control of the secretome and oncogenic mutations seems plausible hypotheses related to chemoresistance generated by the secretome, but additional studies are necessary to confirm them.

It is worth discussing how therapeutic treatments can alter the composition and abundance of the tumor secretome, a phenomenon called “therapy-induced tumor cell secretome,” which enhances the survival and expansion of CSCs [236]. For example, treatment with paclitaxel or gemcitabine in TNBC increases the production of HIF factors, whose increased activity leads to an increase in the CSC subpopulation through IL-6 and IL-8 signaling and increased expression of multidrug resistance. This suggests that a treatment based on chemotherapeutic agents combined with HIF inhibitors would help overcome chemoresistance [23]. However, chemotherapy is not the only treatment that can alter the tumor secretome. As shown in a previous study by our research team, the dose of radiation therapy administered in vitro and in vivo affects the expression of several MMPs and their inhibitors [237]. These alterations were related with the molecular profile of breast cancer. The study not only highlights the fundamental role played by the specific characteristics of each tumor and TME in response to treatment, but also that radiotherapy promotes the secretion of matrix remodeling enzymes involved in the dispersion, invasiveness and metastasis of CSCs and in the EMT process [237]. Additionally, Shen et al. demonstrated that chemotherapy induces breast cancer cells to secrete exosome-derived miR-9, miR-195 and miR-203a, which induces CSC phenotypes and expression of stemness-associated genes, thus generating cancer cell communication and self-adaptation to survive treatment [39]. Thereby, miRNAs can also display chemoprotective functions*,* which means the secretome’s capacity to protect tumors against chemotherapy, as demonstrated by the mentioned studies, in which the released exosomal miRNAs act in response to the treatment favoring the maintenance and expansion of CSCs, avoiding therefore the effect of the treatment and the development of relapses and metastatic processes.

To recapitulate, CSC secretome promotes chemoresistance through different strategies such as inducing the stem phenotype and EMT processes, apoptosis evasion mechanisms and regulation of the immune system. Lastly, chemotherapeutic agents can alter the tumor secretome and consequently tumor cell functions and responses, with a negative effect on treatment outcomes.

## Clinical implications and future trends

**Fig. 3:**
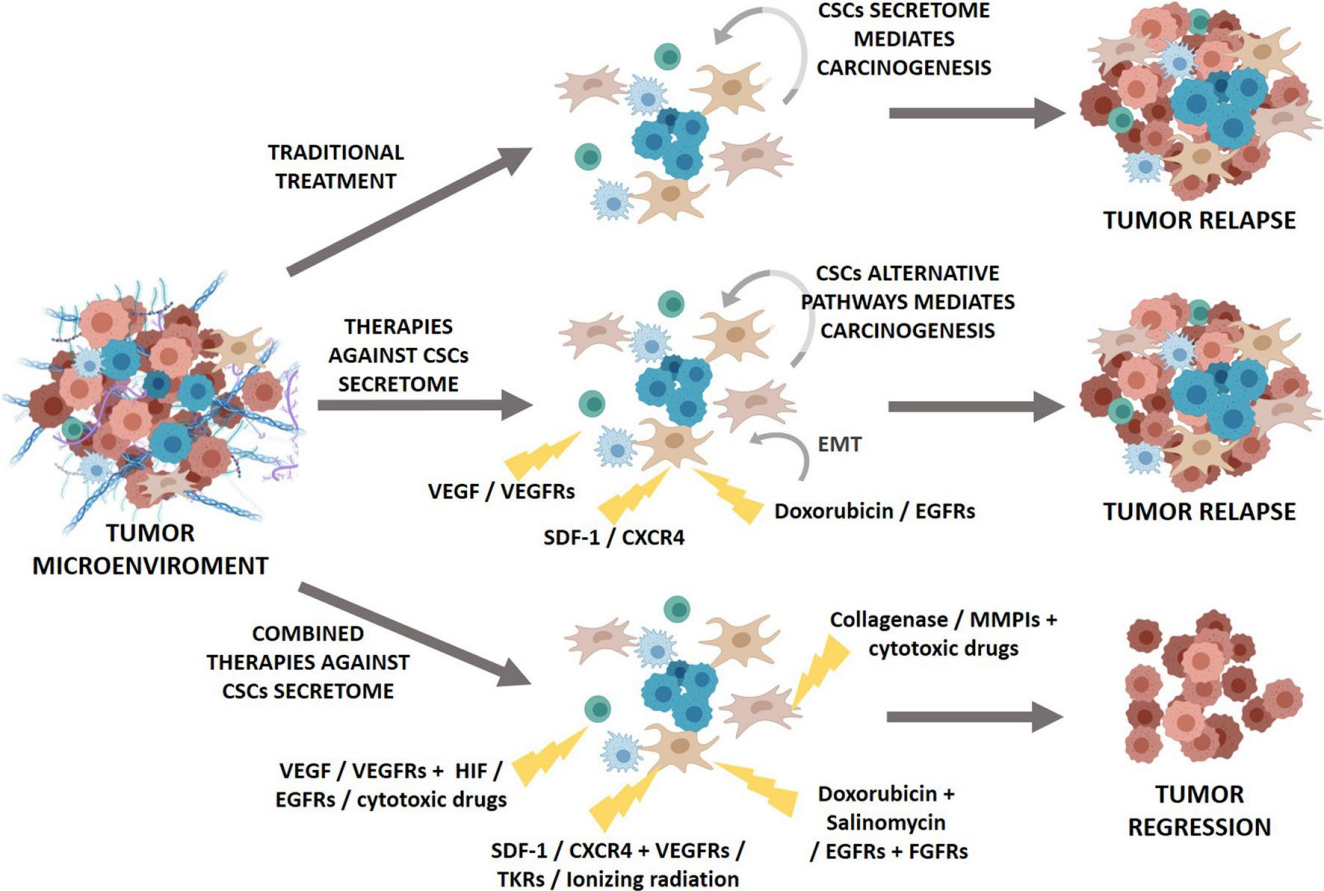
Tumor response to different antitumor strategies. The failure of conventional therapies is due to the tumor and the CSC mechanisms to initiate the carcinogenesis process. For this reason, the new therapies focus on TME, including the CSC secretome. However, CSCs use different pathways to fulfill their functions; therefore, targeting only one of the pathways can lead to tumor relapse. The new therapies are aimed at simultaneously blocking several pathways for better outcomes

Given the importance of the interplay between CSCs and their niche, the new antitumor therapies focus on simultaneously targeting different communication routes to target TME and starve CSCs (Fig. 3). One of the most recurrent options is to target tumor vasculature, with several FDA-approved angiogenesis inhibitors available (see Table 2) such as bevacizumab (antibody directed against VEGF) or sorafenib and sunitinib, inhibitors of tyrosine kinase receptors (TKRs) that target multiple TKRs, including VEGF receptors (VEGFRs) and PDGF receptors (PDGFRs). The combination of both treatment strategies has increased patient survival in the first months, usually in combination with other chemotherapy approaches; however, in many of these patients the disease will progress [238]. This may be due to a lack of biomarkers to determine which patients will benefit from these drugs and the doses required as well as to tumor adaptive resistance mechanisms [239, 240]. This tumor capacity to adapt to therapy by activating other alternative pathways has led to the development of strategies that combine anti-VEGF agents with other drugs targeting different pathways such as VEGFRs, TKRs and epidermal growth factor receptors (EGFRs) inhibitors, with greater or lesser success [241]. Indeed, CSCs can also promote resistance to anti-angiogenic therapy, which leads to intra-tumor hypoxia states resulting in increased HIF-1 and HIF-2 expression and, therefore, increased risk of tumor propagation, CSC self-renewal, drug resistance and even angiogenesis activation [23, 120–122, 125–127]. For example, treatment of breast cancer with sunitinib and bevacizumab increased the CSC population through HIF-1 activation of Wnt pathway [242], and in pancreatic cancer and glioblastoma the use of a VEGFR and TKR inhibitor also increased the risk of invasion and metastasis related to intratumor hypoxic states [243–245]. Nonetheless, when these drugs are used in combination with other cytotoxic drugs, the results are more promising [246, 247], which confirms the idea of using antiangiogenic drugs in conjunction with other therapies for example targeting hypoxia [248] (Fig. 3). Furthermore, antiangiogenic therapy failure has resulted in a different approach involving vascular normalization to improve drug delivery and limit hypoxia [116, 249].

**Table 2:** US FDA-approved secretome targeting drugs

Drug	Target	Cancer type	References
Abiraterone	Androgen deprivation therapy	Prostate cancer	[309]
Aflibercept	Bind VEGF A and B and PGF	Colorectal cancer	[315]
Axitinib	Against VEGR1-3, PDGFRs, c-Kit and FGFRs	Advanced renal cell carcinoma and soft tissue sarcoma	[316, 317]
Bevacizumab	Antibody against vascular endothelial growth factor (VEGF)	Breast, colon and lung cancer	[238]
Cabozantinib	MET and VEGFR2 inhibitor	Renal cancer and hepatocellular carcinoma	[318]
Dacomitinib	EGFRs inhibitor	Metastatic NSCLC	[319]
Enzalutamide	Androgen deprivation therapy	Prostate cancer	[309]
Erdafitinib	FGF receptor (FGFR) inhibitor	Urothelial carcinoma	[320, 321]
Erlotinib	EGFRs inhibitor	NSCLC and pancreatic cancer	[322]
Gefitinib	EGFRs inhibitor	NSCLC	[322]
Lapatinib	EGFRs inhibitor	Breast cancer and NSCLC and pancreatic cancer	[322]
Lenvatinib	Against VEGFR1-3, FGFR1-4, RET, c-kit, and PDGFRα	Thyroid cancer	[323]
Mogamulizumab	Antibody against CCR4	Skin lymphoma	[324]
Oncolytic virus (talimogene laherparepvec)	Expressing GM-CSF to enhance systemic antitumor immune responses	Melanoma	[308]
Osimertinib	EGFRs inhibitor	NSCLC	[322]
Panitumumab	Antibody against endothelial growth factor receptor (EGFR)	Colorectal carcinoma	[325]
Pazopanib	Against VEGR1-3, PDGFRs, c-Kit, and FGFRs	Advanced renal cell carcinoma and soft tissue sarcoma	[316, 317]
Ramucirumab	VEGFR2 inhibitor	Metastatic gastric and gastro-esophageal junction adenocarcinoma	[326]
Regorafenib	TKRs inhibitor, including VEGFR1-3, FGFRs and PDGFRs	Colorectal cancer and hepatocellular carcinoma	[327]
Sorafenib Sunitinib	Tyrosine kinase receptors (TKRs) inhibitors, that target multiple TKRs, including VEGF receptors (VEGFRs) and PDGF receptors (PDGFRs)	Kidney cancer, renal cell carcinoma and gastrointestinal stromal tumors	[238]
Vandetanib	VEGFR2 and EGFR inhibitor	Medullary thyroid carcinoma	[328]

Another widely used approach is to try to block the recruitment or function of stromal cells due to the ability of CSCs to promote their tumor niche. For example, preclinical studies showed that targeting CCL2 or the CCL2 receptor (CCR2) on tumor infiltrating macrophages improved chemotherapeutic efficacy, inhibited metastasis and increased anti-tumor T-cell responses [72, 250, 251]. However, these agents may need to be administered as adjuvant therapy and not as monotherapy [252, 253]. However, the results from clinical trials have not been as promising and a deeper understanding of the underlying mechanisms of the pathways involved is needed in order to success in the clinical translation [254]. A similar result has been observed in treatment with IL-6 inhibitors and its receptor where preclinical trials showed antitumor efficacy against different tumor types, but the clinical trials do not seem to show good results [255]. This suggests the need for biomarkers that may help identify target patients and the need for using combined therapies [256–258]. SDF-1/CXCL12 is another protein related to the recruitment and activation of ECs in TME by CSCs [94], to chemoresistance processes, and trials with inhibitors of SDF-1/CXCL12 or its receptor CXCR4 have shown high antitumor potential [220, 259–262] (Fig. 3). In fact, it is already used in the clinical practice [263]. Moreover, there is evidence of its potential as adjuvant therapy by sensitizing the tumor to other therapies [262, 264–268].

Another strategy used has been to directly attack CSC maintenance. For example, IL-8 antagonists such as reparixin in monotherapy or combined with other drugs have shown their efficacy in in vitro and in vivo assays [149, 269–272]. In fact, ongoing clinical trials demonstrate no adverse effects and good prognosis [273, 274]. Other clinical trials targeting TGF-ß have been carried out, but its pro-tumorigenic effect depends on the tumor stage and the number of signaling pathways where TGF-ß is involved require special attention and robust biomarkers to predict its effect [275].

As previously reported, CSC-secreted factors promote EMT and confer drug resistance, and therefore, many studies have focused on EMT. For example, one of the most studied drugs is salinomycin, which inhibits EMT and sensitizes the tumor to the action of other drugs such as doxorubicin [276–282] (Fig. 3). Metformin also selectively acts against CSCs by targeting EMT, blocking the IL-6/STAT3 axis or decreasing EMT transcriptional factors, and by increasing tumor sensitivity against other current therapies [197, 283–287]; therefore, it has been included in clinical trials [288]. Furthermore, to overcome the resistance acquired by EMT from EGFRs inhibitors, several studies that combine EGFRs inhibitors with drugs targeting other pathways, such as with FGFRs inhibitors, have been performed [289–292]. Despite some good results with some drugs against EMT, their toxicity and ability to promote metastasis still remain a concern [222].

In addition to the previous results, new therapies targeting both the ECM [20, 293] and the MMPs are now emerging [294]. Since one of the main problems is that tumor ECM prevents the correct diffusion of drugs, numerous studies focus on either degrading the matrix, using collagenases, hyaluronidases or hyperthermia [295–300], and on blocking ECM synthesis de novo [301–305], although the latter has not provided positive results in clinical trials [306, 307]. MMPs, which promote angiogenesis, metastasis and the release of factors within the matrix, are also the target of new therapies like andecaliximab and several MMPs antibodies, specific or broad-spectrum MMP inhibitors, which have shown promising results in clinical trials [294] (Fig. 3).

Targeting cancer cell secretome involves not only inhibiting the release or binding with receptors, but rather to stimulate the release of certain factors. For example, talimogene laherparepvec (T-VEC), an oncolytic virus FDA approved for the treatment of advanced melanoma, has been engineered to selectively replicate within tumors and to promote the priming of T cell responses and produce granulocyte–macrophage colony-stimulating factor (GM-CSF) to enhance systemic antitumor immune responses [308]. The role of the tumor secretome in general, and of CSCs in particular, is emerging as an indicator of the response to treatment as shown by the two FDA-approved drugs for androgen deprivation therapy for metastatic prostate cancer, abiraterone and enzalutamide, whose resistance seems to be related to a potentially immunosuppressive tumor microenvironment and whose treatment efficacy can be predicted using IL-6 levels [309].

Finally, a deeper knowledge about the components and dynamics of the CSC secretome has allowed the development of new therapies specifically targeting CSCs through different factors and their overexpressed receptors. In this respect, several clinical trials are conducted to determine the effect of different drugs against CSC secretome and to establish predictive biomarkers for better treatment outcomes (Table 3).

**Table 3:** Clinical trials with CSCs and secretome

NTC number	Target	Status	Drug	Combined therapy	Cancer type
NCT01861054	CXCR1	Completed	Reparixin		Breast cancer
NCT02001974	CXCR1	Phase 1/completed	Reparixin	Paclitaxel	Breast cancer
NCT01190345	VEGF	Phase 2/completed	Bevacizumab	Chemotherapy	Breast cancer
NCT01283945	VEGFR/FGFR/PDGFR	Phase 1/2a completed	Lucitanib		Solid tumor
NCT02491840	CXCR4	Recruiting	Prognostic biomarkers		Gastric and cardia adenocarcinoma
NCT01955460	TGF-ß	Phase 1/recruiting	Aldesleukin	Chemotherapy and lymphocytes	Melanoma
NCT01248637	HIF-1	Completed	Pimonidazole hydrochloride		Pancreatic
NCT04137627	HIF-1	Phase 3/completed	Melatonin	Adjuvant chemotherapy	Oral squamous cell carcinoma
NCT02499458	HIF-2	Completed	Biomarkers		Renal cancer
NCT03401788	HIF-2	Phase 2/not recruiting	PT2977		VHL-associated renal cell carcinoma
NCT03108066	HIF-2	Phase 2/not recruiting	PT2385		VHL-associated renal cell carcinoma
NCT01283945	FGF	Phase ½ completed	Lucitanib		Solid tumors
NCT00657423	FGF	Phase 3	Endostar	Docetaxel and cisplatin	Lung neoplasms
NCT01440959	FGF	Phase 2/completed	Dovitinib		Gastrointestinal stromal tumors
NCT00372775	FGF	Phase 2/completed	Sunitinib		Non-small cell lung cancer with brain metastasis
NCT01791985	FGF	Phase 1 Phase 2 Completed	AZD4547	Anastrozole or letrozole	Breast cancer
NCT01945164	FGF	Completed	XL999		Advanced malignancies
NCT00021229	FGF	Phase 1/2	Imatinib mesylate	Local irradiation therapy	Glioma
NCT04207086	FGF	Phase 2/recruiting	Pembrolizumab Lenvatinib		Melanoma stage III
NCT03303885	FGF	Recruiting	Preclinical biomarkers		Liposarcoma
NCT00216112	PDGF	Phase 2/completed	Matinib, mesylate Docetaxel		Ovarian cancer
NCT03851614	PDGF	Phase 2/recruiting	Cediranib	Durvalumab	Colorectal cancer Pancreatic adenocarcinoma Leiomyosarcoma
NCT01372813	PDGF	Phase 2/completed	Vandetanib		Renal carcinoma
NCT04042597	PDGF	Phase 2/recruiting	Anlotinib hydrochloride		Chordoma advanced cancer
NCT00367679	PDGF	Phase 2/completed	Pazopanib		Non-small cell lung cancer
NCT00372775	PDGF	Phase 2/completed	Sunitinib		Non-small cell lung cancer
NCT01105533	PDGF	Phase 1/completed	PF-00337210		Neoplasm
NCT00600821	PDGF	Phase 2/completed	AG-013736 (axitinib)	Paclitaxel and carboplatin	Non-small cell lung carcinoma
NCT04207086	PDGF	Phase 2/completed	Lenvatinib	Pembrolizumab	Melanoma stage III
NCT02178072	CCL5	Phase 2/recruiting	5-Azacitadine		Head and neck squamous cell carcinoma
NCT03126630	CCL5	Phase 1/2 recruiting	Pembroli Anetumab ravtansinezumab		Pleural malignant mesothelioma
NCT03964337	CCL5	Phase 2	Cabozantinib		Prostate cancer
NCT02125344	CCL5	Phase 3/completed	Chemotherapy		Breast cancer
NCT02432378	CCL5	Phase ½ recruiting	Cisplatin and DC vaccine	Celecoxib CKM	Ovarian cancer
NCT00653250	PEG2	Completed	Celecoxib		Lung cancer

Importantly, as the previous studies have shown, only some patients will effectively benefit from combined therapies while for other patients this type of therapy will be ineffective or even harmful, due to the high heterogeneity between patients. In this respect, several clinical trials are currently conducted to test and validate personalized therapies. A prospective clinical trial that began in 2015 is testing in vitro 73 drugs in different combinations in CSCs from glioblastoma samples to study the effect of personalized therapies [ClinicalTrials.gov identifier: NCT02654964]. Indeed, another prospective clinical trial suggested that personalized antitumor therapies based on molecular profiles of cancer patients had a significant improvement in treatments, compared with current clinical strategies [ClinicalTrials.gov identifier: NCT02534675]. There is increasing evidence that the use of biomarkers would substantially improve clinical practice and patient well-being. Several factors of the CSC secretome have been proposed as biomarkers, like ceruloplasmin identified in pancreatic cancer, which could be used in addition to CA19-9 [310]. Ceruloplasmin was also identified in serum from ovarian cancer patients as a possible prognostic biomarker of chemoresistance [311]. Its overexpression by CSCs in glioma has also been studied, but currently only in vitro and in vivo [312]. In blood samples from breast cancer patients, CSC-secreted programmed death ligand-1 (PD-L1) was related with metastasis and has been proposed as a potential follow-up biomarker by immune checkpoint blockers [313]. However, finding robust markers can be challenging. As for antiangiogenic therapies, numerous biomarkers have been proposed since the approval of bevacizumab, being VEGF-A the most promising. However, in clinical trials its efficacy could not be proven [314] and more research is still needed to identify predictive and prognostic biomarkers.

## Conclusion

**Fig. 4:**
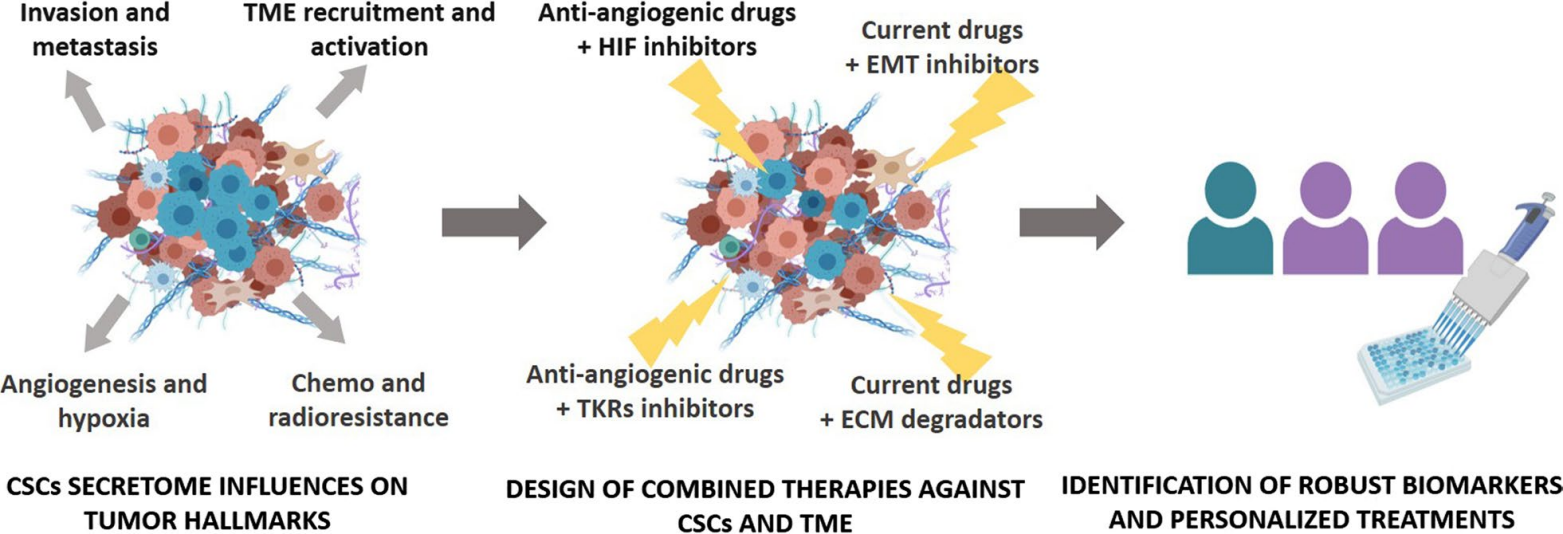
Development of new personalized antitumor therapies for cancer patients. Due to the importance of CSC secretome in tumor development, chemoresistance and relapse, the new therapies need to combine different TME and CSC inhibitors. Robust biomarkers are required to identify the patients that will benefit from these treatments

The evidence included in this review demonstrates that CSCs regulate multiple tumor hallmarks through the expression of several growth factors, interleukins, cytokines and extracellular vesicles, and that a greater understanding of the pathways that dictate tumor behavior is needed for the development of new antitumor therapies. Furthermore, these therapies must target not only tumor proliferating cells and CSCs, but also they need to be combined with other therapies targeting TME. The interconnected signaling pathways involved in the altered secretome must also be targeted, since tumors can develop evasion mechanisms and have different alternative routes to fulfil their functions. Lastly, robust biomarkers are needed to identify those patients most likely to benefit from these therapies in order to personalize antitumor treatments (Fig. 4).

## Data Availability

Not applicable.

## Abbreviations

ALDH: Aldehyde dehydrogenase
BMDCs: Bone marrow-derived cells
CAFs: Cancer-associated fibroblasts
CLIC1: Chloride intracellular channel protein 1
CSCs: Cancer stem cells
CTGF: Connective tissue growth factor
ECs: Endothelial cells
ECM: Extracellular matrix
EGFRs: Epidermal growth factor receptors
EMT: Epithelial–mesenchymal–transition
EPCs: Endothelial progenitor cells
FGF: Fibroblast growth factor
GSCs: Glioblastoma stem-like cells
HGF: Hepatocyte growth factor
Hh: Hedgehog
HIF-1: Hypoxia-inducible factor 1
HIF-2: Hypoxia-inducible factor 1
HDGF: Hepatoma-derived growth factor
IGF-1: Insulin-like growth factor 1
IGF1R: Insulin-like growth factor 1 receptor
IL-1ß: Interleukin 1-ß
IL-2: Interleukin 2
IL-4: Interleukin 4
IL-6: Interleukin 6
IL-8: Interleukin 8
IL-10: Interleukin 10
LIF: Leukemia inhibitory factor
MIC-1: Macrophage inhibitory cytokine 1
miRNAs: Micro-RNAs
MMPs: Matrix metalloproteinases
mRNAs: Messenger RNAs
MSCs: Mesenchymal stem cells
MVs: Microvesicles
NF-kB: Nuclear factor kappa B
PAR1: Protease-activated receptor 1
PDGF: Platelet-derived growth factor
PDGFRs: Platelet-derived growth factor receptors
PD-L1: Programmed death ligand 1
PGE2: Prostaglandin E2
PMN: Premetastatic niche
SC: Stem cell
SDF-1/CXCL12: Stromal cell-derived factor 1
SHH: Sonic Hedgehog
TAMs: Tumor-associated macrophage
TGF-ß: Transforming growth factor ß
TKRs: Tyrosine kinase receptors
TME: Tumor microenvironment
TNBC: Triple-negative breast cancer
Treg: Regulatory T lymphocytes
VE-Cadherin: Vascular–endothelial cadherin
VEGF: Vascular–endothelial growth factor
VEGFR: Vascular–endothelial growth factor receptor
